# Supplementary material for: Transcriptome-Wide Analysis Reveals Key DEGs in Flower Color Regulation of Hosta plantaginea (Lam.) Aschers
Source: Genes (Basel). 2019 Dec 26;11(1):31. doi: 10.3390/genes11010031 (PMC7017146; doi:10.3390/genes11010031)
Supplement: Supplementary file 1 [file genes-11-00031-s001.zip › supplementary materials-1.pdf]

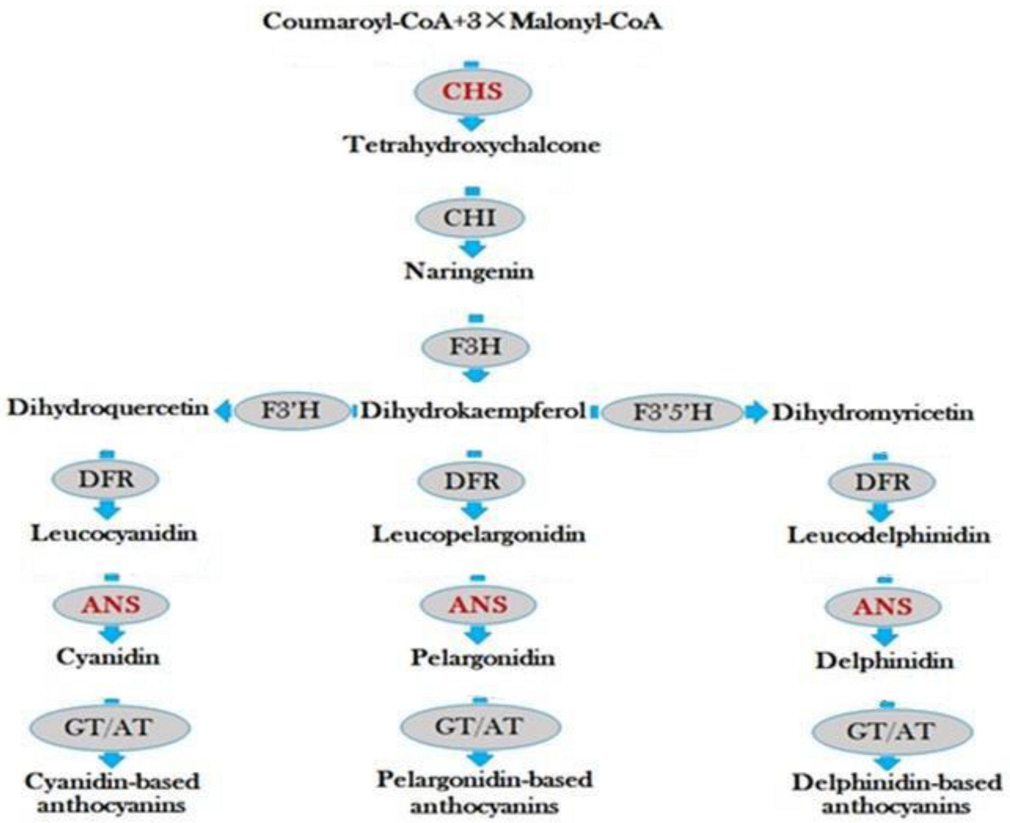

Figure S1. Anthocyanin biosynthesis pathway

The first stage is the phenylpropane pathway, and transforms into coumarin CoA form phenylalanine through three-step enzymatic reaction, phenylalaninammo-nialyase (PAL) as the key enzyme in this stage. The second stage, from coumarin CoA to dihydroflavonol, is the key reaction of flavonoid metabolism, the main catalytic enzymes include chalcone synthase, chalcone isomerase and Flavanone-3-hydroxylase. The third stage is the synthesis of various anthocyanins, dihydroflavonols catalyzed by flavonoid -3'-hydroxylase and flavonoid -3',5'-hydroxylase can be converted into colorless anthocyanins under the action of dihydroflavonol 4-reductase gene. At the last stage, colorless anthocyanins are catalyzed by anthocyanidinsynthase to form colored but unstable anthocyanins, and the catalytic action of UDP glucose-flavonoid- 3-o-glycosyltranferase makes anthocyanins become stable anthocyanins.

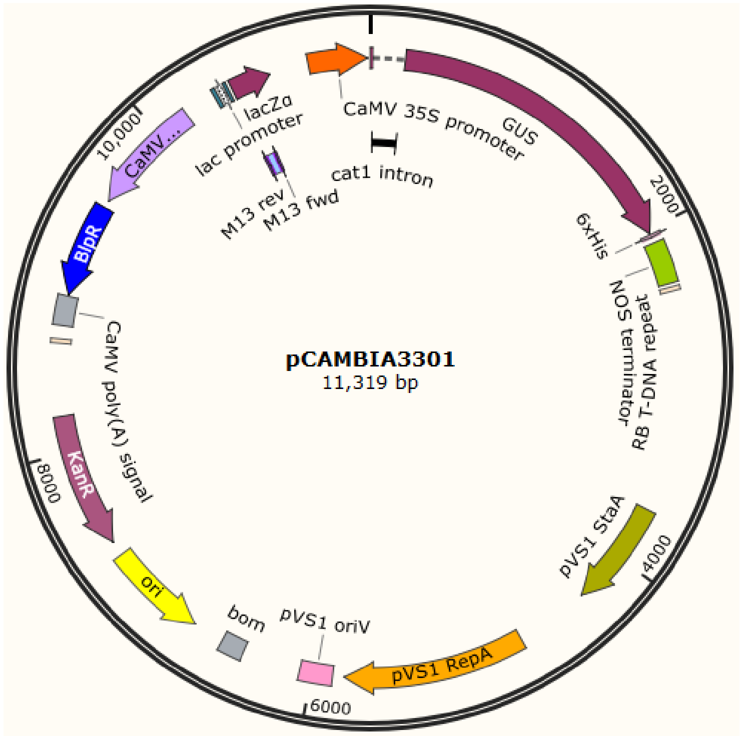

Figure S2. The expression vector pCAMBIA3301is preserved by Changbai mountain characteristic plant resources research laboratory of jilin agricultural university.

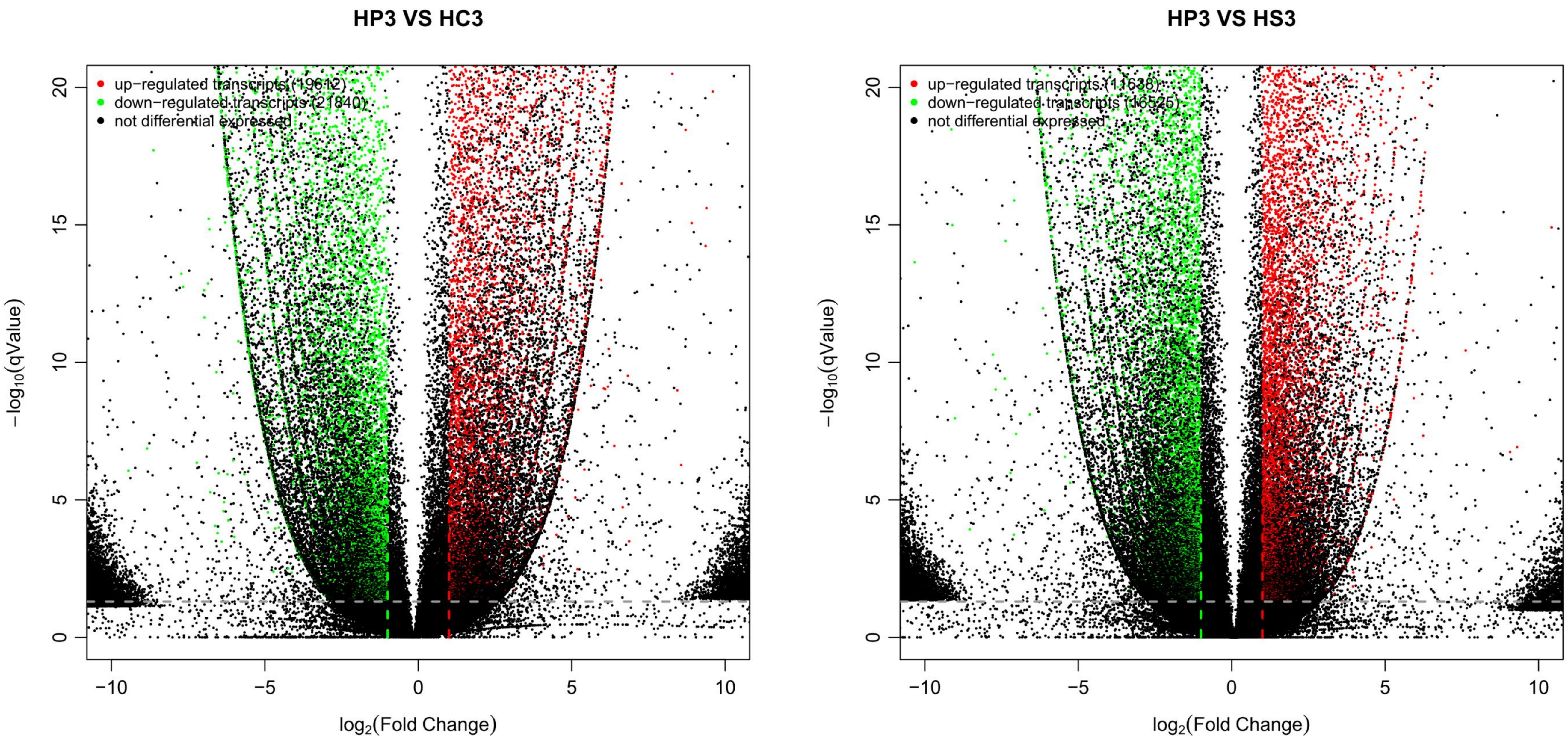

Figure S3. Transcript_volcano of HP3_vs_HC3＆HP3_vs_HS3.

The horizontal axis is the fold-change (log (B/A)) value of the differential expression of the gene in different groups of samples, and the vertical axis is the statistical significance level pValue representing the change in gene expression, the smaller the pValue, the -log (pValue) The bigger the difference, the more significant the difference. Each point in the figure represents a gene in which red indicates up-regulated genes, green indicates down-regulated genes, and black indicates non-differentiated genes.

Table S1. Primers for qRT-PCR

gene	primer 5′-3′
c56407-F	5'ACCAGGCAATGCTATGAACAGA'3
c56407-R	5'TGCTGTAGATTCCCCTCGCT'3
c141633-F	5'GTCAGCCAGAGCGCGGTATTG'3
c141633-R	5'AGTTCTTCAGCCAGGATGCCATTG'3
c128205-F	5'GCTCCGAGGTGCTCACTGATAATG'3
c128205-R	5'TGCCGACTCTCCGCCTTACG'3
c125088-F	5'GCGGTGAAGATGTGGACGAAGC'3
c125088-R	5'CCTGGTGCCGAGTATGCGAATAAC'3
c134145-F	5'GGCTGGCTGCATTGCTTAGA'3
c134145-R	5'CAATGTCAGACCTCACCCGC'3
CHS-F	5'TTCAAGCCGTTGGGGATCTC'3
CHS-R	5'ACTCAGCACTTGTCTCGTGG'3
ANS-F	5'GAGTGTGCGGAGAGTGTCAA'3
ANS-R	5'TCGGGAGATCGAAGAACCCT'3
P450-c120890-F	5'GGCATGGCTCACTTCTCCTGTTG'3
P450-c120890-R	5'TGCCGCTCGGAAGTACCTCAG'3
HvActin-F	5'AACCACCTTAATCTTCATGCTGCT'3
HvActin-R	5'AAGATTCAGATGCCCAGAGGTCCT'3

Table S2. GO, KEGG, and KOG enrichment.

KEGG-id	Description	Significant	Annotated	Genes involved in the pathway	*p* value
ko01200	Carbon metabolism	134 /2030	303 /5870	TRINITY_DN66055_c1_g1;TRINITY_DN71804_c0_g3;TRINITY_DN79527_c1_g2;TRINITY_DN78257_c1_g2;TRINITY_DN54898_c0_g1;TRINITY_DN75166_c1_g1;TRINITY_DN79467_c1_g2(down);TRINITY_DN87258_c0_g1;TRINITY_DN58754_c0_g2;TRINITY_DN76907_c1_g2;TRINITY_DN79925_c4_g3;TRINITY_DN79925_c4_g2;TRINITY_DN51154_c1_g1;TRINITY_DN78109_c1_g1;TRINITY_DN76548_c2_g1;TRINITY_DN67581_c1_g1(down);TRINITY_DN83109_c3_g1(up);TRINITY_DN52168_c0_g2;TRINITY_DN80257_c2_g1(down);TRINITY_DN77599_c0_g3(down);TRINITY_DN71838_c1_g1;TRINITY_DN76475_c1_g2(up);TRINITY_DN67359_c0_g1;TRINITY_DN77567_c0_g2;TRINITY_DN67313_c0_g1(down);TRINITY_DN71433_c3_g1;TRINITY_DN67579_c0_g1;TRINITY_DN68783_c2_g3;TRINITY_DN72896_c0_g2(down);TRINITY_DN66911_c1_g1;TRINITY_DN79131_c0_g3;TRINITY_DN63538_c4_g1;TRINITY_DN55308_c0_g2(up);TRINITY_DN104807_c0_g1;TRINITY_DN66699_c0_g1;TRINITY_DN69730_c1_g4;TRINITY_DN78109_c0_g1;TRINITY_DN68189_c0_g3(up);TRINITY_DN76773_c1_g3;TRINITY_DN77891_c0_g1;TRINITY_DN55506_c8_g1(up);TRINITY_DN80650_c1_g2;TRINITY_DN74721_c0_g1;TRINITY_DN50551_c0_g1;TRINITY_DN71600_c1_g2;TRINITY_DN78994_c1_g1(down);TRINITY_DN76269_c0_g2(up);TRINITY_DN74067_c0_g1;TRINITY_DN67579_c0_g3;TRINITY_DN69110_c0_g4(up);TRINITY_DN79527_c1_g1;TRINITY_DN69291_c2_g2;TRINITY_DN52045_c0_g2(down);TRINITY_DN70487_c4_g2(up);TRINITY_DN74270_c0_g1;TRINITY_DN62026_c1_g2(down);TRINITY_DN76699_c2_g1;TRINITY_DN76699_c2_g2;TRINITY_DN82845_c5_g2;TRINITY_DN80208_c5_g1(down);TRINITY_DN47629_c0_g1;TRINITY_DN78755_c1_g1(down);TRINITY_DN76791_c0_g1;TRINITY_DN49511_c0_g1;TRINITY_DN53805_c0_g1(up);TRINITY_DN79267_c3_g1;TRINITY_DN76269_c0_g3;TRINITY_DN73371_c1_g1;TRINITY_DN67492_c0_g2(up);TRINITY_DN77567_c0_g1;TRINITY_DN61815_c0_g2(up);TRINITY_DN60378_c0_g1;TRINITY_DN69338_c0_g1;TRINITY_DN71462_c0_g2;TRINITY_DN60864_c1_g1(down);TRINITY_DN79901_c2_g1;TRINITY_DN82998_c8_g2;TRINITY_DN77897_c1_g1(up);TRINITY_DN76525_c0_g6;TRINITY_DN75460_c3_g1;TRINITY_DN54601_c1_g1;TRINITY_DN79587_c1_g1;TRINITY_DN79065_c1_g1;TRINITY_DN78201_c1_g3;TRINITY_DN62470_c1_g1;TRINITY_DN62681_c0_g1;TRINITY_DN78048_c1_g1;TRINITY_DN66212_c2_g2;TRINITY_DN60691_c1_g4;TRINITY_DN61342_c1_g2(down);TRINITY_DN78116_c2_g3;TRINITY_DN77851_c0_g1;TRINITY_DN82434_c2_g2(down);TRINITY_DN67108_c0_g1;TRINITY_DN108510_c0_g1;TRINITY_DN55174_c1_g4;TRINITY_DN68562_c0_g2;TRINITY_DN71321_c0_g2;TRINITY_DN74786_c2_g3;TRINITY_DN68004_c2_g2(up);TRINITY_DN78782_c2_g1(up);TRINITY_DN52789_c0_g1;TRINITY_DN52435_c0_g1;TRINITY_DN72897_c0_g3;TRINITY_DN82682_c2_g2(down);TRINITY_DN77290_c2_g1;TRINITY_DN70284_c0_g1(down);TRINITY_DN76573_c2_g2;TRINITY_DN70178_c0_g1;TRINITY_DN31309_c0_g1;TRINITY_DN78620_c0_g1(down);TRINITY_DN48647_c0_g1;TRINITY_DN58382_c0_g1;TRINITY_DN79817_c2_g1;TRINITY_DN72759_c1_g1(down);TRINITY_DN60680_c2_g2;TRINITY_DN60649_c1_g4;TRINITY_DN79763_c2_g3;TRINITY_DN82649_c2_g2(down);TRINITY_DN79638_c3_g2;TRINITY_DN79684_c1_g1(down);TRINITY_DN81896_c4_g4;TRINITY_DN64356_c1_g3;TRINITY_DN76548_c3_g1;TRINITY_DN79505_c0_g1;TRINITY_DN53754_c0_g1(down);TRINITY_DN78370_c1_g1;TRINITY_DN71959_c0_g1;TRINITY_DN82446_c3_g7;TRINITY_DN81944_c3_g1(up);TRINITY_DN72296_c1_g4;TRINITY_DN79384_c2_g1;TRINITY_DN69810_c2_g1;TRINITY_DN74175_c1_g2(down);TRINITY_DN80182_c0_g2;TRINITY_DN77591_c1_g1(down);TRINITY_DN49849_c0_g1;TRINITY_DN80088_c1_g1;TRINITY_DN51329_c1_g2(down);TRINITY_DN69166_c1_g3(up);TRINITY_DN69709_c1_g1(down);TRINITY_DN77716_c1_g1(down);TRINITY_DN80035_c2_g1;TRINITY_DN50455_c0_g1;TRINITY_DN79505_c1_g2;TRINITY_DN81296_c4_g1;TRINITY_DN70734_c0_g2(up);TRINITY_DN75105_c1_g6(up);TRINITY_DN82940_c6_g1;TRINITY_DN76525_c0_g2;TRINITY_DN69085_c0_g2;TRINITY_DN63963_c0_g3;TRINITY_DN76712_c0_g1;TRINITY_DN73406_c0_g2(up);TRINITY_DN79918_c2_g2(down);TRINITY_DN76040_c0_g2;TRINITY_DN67443_c2_g3(down);TRINITY_DN78201_c1_g2;TRINITY_DN78703_c0_g3(down);TRINITY_DN73271_c0_g2;TRINITY_DN79505_c2_g1;TRINITY_DN66699_c0_g2(down);TRINITY_DN72609_c1_g1;TRINITY_DN78528_c1_g3(down);TRINITY_DN53097_c0_g2;TRINITY_DN65389_c2_g2;TRINITY_DN72977_c0_g2(up);TRINITY_DN82649_c1_g1;TRINITY_DN61264_c2_g5;TRINITY_DN62685_c2_g1;TRINITY_DN70949_c0_g1;TRINITY_DN53431_c0_g1;TRINITY_DN74536_c0_g1;TRINITY_DN47469_c0_g1;TRINITY_DN71719_c2_g1;TRINITY_DN58377_c0_g1;TRINITY_DN77031_c2_g2;TRINITY_DN82446_c3_g3(up);TRINITY_DN52951_c0_g1;TRINITY_DN78236_c4_g1(down);TRINITY_DN77044_c5_g2(down);TRINITY_DN77827_c1_g1;TRINITY_DN54765_c7_g1;TRINITY_DN82322_c1_g2(up);TRINITY_DN75482_c0_g1;TRINITY_DN69067_c1_g1;TRINITY_DN80010_c1_g1;TRINITY_DN78834_c1_g1(up);TRINITY_DN75611_c1_g2(up);TRINITY_DN60071_c3_g1;TRINITY_DN64613_c0_g3;TRINITY_DN80426_c1_g1;TRINITY_DN78427_c3_g3;TRINITY_DN69414_c0_g1;TRINITY_DN81443_c2_g3;TRINITY_DN81443_c2_g2;TRINITY_DN75150_c1_g1;TRINITY_DN71003_c0_g1;TRINITY_DN54851_c1_g1;TRINITY_DN62908_c0_g1;TRINITY_DN82316_c4_g2;TRINITY_DN47023_c0_g1;TRINITY_DN76525_c0_g3;TRINITY_DN64472_c0_g1;TRINITY_DN75606_c1_g4;TRINITY_DN72897_c0_g1;TRINITY_DN58641_c2_g1;TRINITY_DN76864_c0_g2(down);TRINITY_DN74148_c0_g1;TRINITY_DN67492_c0_g3;TRINITY_DN79053_c2_g1;TRINITY_DN77851_c3_g1;TRINITY_DN79791_c1_g1;TRINITY_DN56846_c0_g1;TRINITY_DN76355_c1_g1;TRINITY_DN63572_c0_g1(up);TRINITY_DN78957_c0_g5;TRINITY_DN69451_c0_g2;TRINITY_DN74911_c0_g4(up);TRINITY_DN78778_c0_g2;TRINITY_DN65603_c4_g1;TRINITY_DN70178_c0_g2;TRINITY_DN65431_c0_g1;TRINITY_DN71378_c0_g1;TRINITY_DN76773_c1_g2;TRINITY_DN78589_c1_g1(up);TRINITY_DN62132_c3_g1;TRINITY_DN69951_c0_g10;TRINITY_DN54642_c2_g1;TRINITY_DN79107_c1_g1;TRINITY_DN69134_c0_g3;TRINITY_DN55937_c0_g1;TRINITY_DN64645_c1_g2;TRINITY_DN62265_c2_g1;TRINITY_DN50123_c0_g1(up);TRINITY_DN79031_c0_g1;TRINITY_DN78276_c2_g3;TRINITY_DN78276_c2_g2;TRINITY_DN77681_c2_g2;TRINITY_DN67049_c0_g1;TRINITY_DN77681_c2_g5;TRINITY_DN71370_c0_g2;TRINITY_DN78276_c2_g4;TRINITY_DN79467_c1_g1(up);TRINITY_DN78933_c0_g1;TRINITY_DN74666_c1_g2;TRINITY_DN60374_c0_g3;TRINITY_DN66759_c1_g1;TRINITY_DN81830_c2_g3;TRINITY_DN83048_c2_g1(down);TRINITY_DN80180_c2_g1(down);TRINITY_DN68313_c1_g2;TRINITY_DN79600_c3_g1;TRINITY_DN75777_c1_g1(down);TRINITY_DN65027_c0_g1;TRINITY_DN62416_c0_g1;TRINITY_DN65162_c0_g2;TRINITY_DN75599_c0_g1;TRINITY_DN70907_c1_g2;TRINITY_DN65875_c1_g1;TRINITY_DN77993_c5_g1;TRINITY_DN57077_c1_g3;TRINITY_DN70230_c0_g1;TRINITY_DN59441_c0_g1;TRINITY_DN80153_c1_g2;TRINITY_DN72574_c2_g1;TRINITY_DN80629_c3_g1(up);TRINITY_DN80874_c4_g4;TRINITY_DN79920_c1_g2;TRINITY_DN46019_c0_g1;TRINITY_DN79424_c1_g1(up);TRINITY_DN82150_c4_g3(up);TRINITY_DN79165_c2_g1(down);TRINITY_DN75165_c0_g1;TRINITY_DN69810_c2_g2;TRINITY_DN69951_c0_g5;TRINITY_DN66656_c1_g2;TRINITY_DN78709_c2_g1;TRINITY_DN70349_c3_g3;TRINITY_DN74016_c1_g1(up);TRINITY_DN79388_c2_g2;TRINITY_DN73019_c0_g1(down);TRINITY_DN53354_c0_g1(up);TRINITY_DN80037_c5_g1;TRINITY_DN62028_c1_g6;TRINITY_DN68321_c0_g1;TRINITY_DN77259_c1_g1;TRINITY_DN70270_c3_g2;TRINITY_DN81176_c2_g3;TRINITY_DN51154_c0_g2;TRINITY_DN79026_c0_g2(down);TRINITY_DN74338_c1_g1(down);TRINITY_DN74175_c1_g1;TRINITY_DN54852_c0_g3;TRINITY_DN72893_c1_g2;TRINITY_DN66990_c1_g1(down);TRINITY_DN60770_c8_g1(down);TRINITY_DN64837_c1_g2;TRINITY_DN71185_c2_g1;TRINITY_DN77296_c2_g2;TRINITY_DN76010_c0_g1;TRINITY_DN57931_c0_g1(down);TRINITY_DN65855_c0_g2(up);TRINITY_DN70589_c0_g3(down);TRINITY_DN65597_c0_g2(up);TRINITY_DN77296_c2_g1(down);TRINITY_DN78406_c1_g2	0.000226359
ko00020	Citrate cycle (TCA cycle)	42 /2030	81 /5870	TRINITY_DN76475_c1_g2(up);TRINITY_DN65389_c2_g2;TRINITY_DN52168_c0_g2;TRINITY_DN62685_c2_g1;TRINITY_DN75166_c1_g1;TRINITY_DN71600_c1_g2;TRINITY_DN78116_c2_g3;TRINITY_DN82434_c2_g2(down);TRINITY_DN58754_c0_g2;TRINITY_DN77567_c0_g2;TRINITY_DN79925_c4_g3;TRINITY_DN79925_c4_g2;TRINITY_DN61342_c1_g2(down);TRINITY_DN53097_c0_g2;TRINITY_DN73406_c0_g2(up);TRINITY_DN74911_c0_g4(up);TRINITY_DN77259_c1_g1;TRINITY_DN79920_c1_g2;TRINITY_DN66990_c1_g1(down);TRINITY_DN82446_c3_g7;TRINITY_DN50123_c0_g1(up);TRINITY_DN81830_c2_g3;TRINITY_DN80180_c2_g1(down);TRINITY_DN70734_c0_g2(up);TRINITY_DN82446_c3_g3(up);TRINITY_DN78620_c0_g1(down);TRINITY_DN78257_c1_g2;TRINITY_DN73216_c1_g1(up);TRINITY_DN48397_c0_g1;TRINITY_DN64629_c1_g2(down);TRINITY_DN58382_c0_g1;TRINITY_DN67579_c0_g1;TRINITY_DN77031_c2_g2;TRINITY_DN67579_c0_g3;TRINITY_DN54765_c7_g1;TRINITY_DN78994_c1_g1(down);TRINITY_DN55308_c0_g2(up);TRINITY_DN104807_c0_g1;TRINITY_DN53754_c0_g1(down);TRINITY_DN79638_c3_g2;TRINITY_DN59441_c0_g1;TRINITY_DN64645_c1_g2;TRINITY_DN72574_c2_g1;TRINITY_DN80629_c3_g1(up);TRINITY_DN52435_c0_g1;TRINITY_DN46019_c0_g1;TRINITY_DN79424_c1_g1(up);TRINITY_DN50551_c0_g1;TRINITY_DN60649_c1_g4;TRINITY_DN76525_c0_g6;TRINITY_DN50304_c1_g1;TRINITY_DN78370_c1_g1;TRINITY_DN69810_c2_g2;TRINITY_DN47023_c0_g1;TRINITY_DN76525_c0_g3;TRINITY_DN72296_c1_g4;TRINITY_DN77290_c2_g1;TRINITY_DN69110_c0_g4(up);TRINITY_DN69810_c2_g1;TRINITY_DN62028_c1_g6;TRINITY_DN68321_c0_g1;TRINITY_DN73019_c0_g1(down);TRINITY_DN80847_c2_g1;TRINITY_DN69414_c0_g1;TRINITY_DN71433_c3_g1;TRINITY_DN82845_c5_g2;TRINITY_DN63880_c1_g1(up);TRINITY_DN82505_c5_g1;TRINITY_DN82682_c2_g2(down);TRINITY_DN80035_c2_g1;TRINITY_DN77567_c0_g1;TRINITY_DN69338_c0_g1;TRINITY_DN78778_c0_g2;TRINITY_DN65603_c4_g1;TRINITY_DN82940_c6_g1;TRINITY_DN76525_c0_g2;TRINITY_DN71462_c0_g2;TRINITY_D	0.000968655

				N78589_c1_g1(up);TRINITY_DN62132_c3_g1;TRINITY_DN81176_c2_g3;TRINITY_DN63963_c0_g3	
ko00680	Methane metabolism	31 /2030	57 /5870	TRINITY_DN78201_c1_g2;TRINITY_DN78201_c1_g3;TRINITY_DN71838_c1_g1;TRINITY_DN69291_c2_g2;TRINITY_DN78048_c1_g1;TRINITY_DN65162_c0_g2;TRINITY_DN78427_c3_g3;TRINITY_DN87258_c0_g1;TRINITY_DN79031_c0_g1;TRINITY_DN79791_c1_g1;TRINITY_DN77681_c2_g2;TRINITY_DN51154_c1_g1;TRINITY_DN68562_c0_g2;TRINITY_DN68004_c2_g2(up);TRINITY_DN74786_c2_g3;TRINITY_DN69451_c0_g2;TRINITY_DN72893_c1_g2;TRINITY_DN82649_c1_g1;TRINITY_DN69134_c0_g3;TRINITY_DN60374_c0_g3;TRINITY_DN76573_c2_g2;TRINITY_DN67359_c0_g1;TRINITY_DN78236_c4_g1(down);TRINITY_DN79817_c2_g1;TRINITY_DN64837_c1_g2;TRINITY_DN82322_c1_g2(up);TRINITY_DN69067_c1_g1;TRINITY_DN78834_c1_g1(up);TRINITY_DN76269_c0_g3;TRINITY_DN75611_c1_g2(up);TRINITY_DN64613_c0_g3;TRINITY_DN79165_c2_g1(down);TRINITY_DN75165_c0_g1;TRINITY_DN76269_c0_g2(up);TRINITY_DN76699_c2_g1;TRINITY_DN81944_c3_g1(up);TRINITY_DN79527_c1_g1;TRINITY_DN79527_c1_g2;TRINITY_DN82649_c2_g2(down);TRINITY_DN80182_c0_g2;TRINITY_DN49849_c0_g1;TRINITY_DN80088_c1_g1;TRINITY_DN76699_c2_g2;TRINITY_DN60071_c3_g1;TRINITY_DN51154_c0_g2;TRINITY_DN76355_c1_g1;TRINITY_DN77716_c1_g1(down);TRINITY_DN79267_c3_g1;TRINITY_DN55174_c1_g4;TRINITY_DN62026_c1_g2(down);TRINITY_DN66759_c1_g1;TRINITY_DN60680_c2_g2;TRINITY_DN76010_c0_g1;TRINITY_DN62265_c2_g1;TRINITY_DN78406_c1_g2;TRINITY_DN75460_c3_g1;TRINITY_DN77681_c2_g5	0.001617087
ko00625	Chloroalkane and chloroalkene degradation	9 /2030	11 /5870	TRINITY_DN71746_c0_g1(down);TRINITY_DN76573_c2_g2;TRINITY_DN78031_c0_g4(down);TRINITY_DN50774_c0_g1(down);TRINITY_DN78236_c4_g1(down);TRINITY_DN80144_c3_g1;TRINITY_DN78031_c0_g1(up);TRINITY_DN78031_c0_g3;TRINITY_DN79840_c3_g1(down);TRINITY_DN71942_c1_g1;TRINITY_DN69134_c0_g3	0.001833013
ko00010	Glycolysis / Gluconeogenesis	63 /2030	136 /5870	TRINITY_DN79845_c3_g4(down);TRINITY_DN71600_c1_g2;TRINITY_DN58754_c0_g2;TRINITY_DN79925_c4_g3;TRINITY_DN79925_c4_g2;TRINITY_DN78031_c0_g3;TRINITY_DN78031_c0_g4(down);TRINITY_DN67359_c0_g1;TRINITY_DN71942_c1_g1;TRINITY_DN64837_c1_g2;TRINITY_DN64125_c4_g1;TRINITY_DN78109_c0_g1;TRINITY_DN68189_c0_g3(up);TRINITY_DN80650_c1_g2;TRINITY_DN50551_c0_g1;TRINITY_DN76269_c0_g2(up);TRINITY_DN50774_c0_g1(down);TRINITY_DN77196_c1_g1;TRINITY_DN79527_c1_g1;TRINITY_DN79527_c1_g2;TRINITY_DN71433_c3_g1;TRINITY_DN68483_c7_g1;TRINITY_DN76355_c1_g1;TRINITY_DN79267_c3_g1;TRINITY_DN74343_c3_g5(up);TRINITY_DN71462_c0_g2;TRINITY_DN60864_c1_g1(down);TRINITY_DN78117_c0_g1;TRINITY_DN82998_c8_g2;TRINITY_DN66949_c0_g1;TRINITY_DN78117_c0_g2;TRINITY_DN52169_c0_g2;TRINITY_DN63963_c0_g3;TRINITY_DN71838_c1_g1;TRINITY_DN78116_c2_g3;TRINITY_DN67108_c0_g1;TRINITY_DN68562_c0_g2;TRINITY_DN74786_c2_g3;TRINITY_DN68004_c2_g2(up);TRINITY_DN63488_c0_g1;TRINITY_DN72897_c0_g1;TRINITY_DN76573_c2_g2;TRINITY_DN74343_c3_g1;TRINITY_DN79817_c2_g1;TRINITY_DN72759_c1_g1(down);TRINITY_DN79763_c2_g3;TRINITY_DN78703_c0_g3(down);TRINITY_DN79638_c3_g2;TRINITY_DN76269_c0_g3;TRINITY_DN52435_c0_g1;TRINITY_DN78370_c1_g1;TRINITY_DN79901_c2_g1;TRINITY_DN50123_c0_g1(up);TRINITY_DN80928_c3_g3;TRINITY_DN65875_c1_g1;TRINITY_DN70734_c0_g2(up);TRINITY_DN82940_c6_g1;TRINITY_DN76525_c0_g2;TRINITY_DN61406_c0_g2;TRINITY_DN76040_c0_g2;TRINITY_DN79706_c2_g3;TRINITY_DN79840_c3_g1(down);TRINITY_DN78236_c4_g1(down);TRINITY_DN61264_c2_g5;TRINITY_DN69134_c0_g3;TRINITY_DN69599_c0_g1(down);TRINITY_DN56693_c1_g1;TRINITY_DN73268_c0_g1;TRINITY_DN79107_c1_g1;TRINITY_DN78031_c0_g1(up);TRINITY_DN82322_c1_g2(up);TRINITY_DN77891_c0_g1;TRINITY_DN78834_c1_g1(up);TRINITY_DN80874_c4_g4;TRINITY_DN64613_c0_g3;TRINITY_DN63361_c0_g1;TRINITY_DN83048_c2_g1(down);TRINITY_DN76525_c0_g6;TRINITY_DN72092_c3_g1;TRINITY_DN47023_c0_g1;TRINITY_DN76525_c0_g3;TRINITY_DN74456_c0_g1(up);TRINITY_DN79093_c1_g2(up);TRINITY_DN72791_c1_g2;TRINITY_DN80010_c1_g1;TRINITY_DN74721_c0_g1;TRINITY_DN60071_c3_g1;TRINITY_DN62774_c1_g1(up);TRINITY_DN78778_c0_g2;TRINITY_DN70178_c0_g1;TRINITY_DN70178_c0_g2;TRINITY_DN60680_c2_g2;TRINITY_DN71378_c0_g1;TRINITY_DN75611_c1_g2(up);TRINITY_DN67049_c0_g1;TRINITY_DN56829_c2_g1;TRINITY_DN82668_c4_g1(up);TRINITY_DN78933_c0_g1;TRINITY_DN79679_c2_g1(down);TRINITY_DN78427_c3_g3;TRINITY_DN75757_c0_g1;TRINITY_DN79031_c0_g1;TRINITY_DN58268_c0_g2;TRINITY_DN77681_c2_g2;TRINITY_DN77400_c0_g1;TRINITY_DN77681_c2_g5;TRINITY_DN69823_c1_g1;TRINITY_DN74814_c0_g3;TRINITY_DN74814_c0_g2;TRINITY_DN77993_c5_g1;TRINITY_DN65162_c0_g2;TRINITY_DN80144_c3_g1;TRINITY_DN70230_c0_g1;TRINITY_DN78109_c1_g1;TRINITY_DN71746_c0_g1(down);TRINITY_DN80629_c3_g1(up);TRINITY_DN79920_c1_g2;TRINITY_DN46019_c0_g1;TRINITY_DN72897_c0_g3;TRINITY_DN48814_c0_g1(up);TRINITY_DN75165_c0_g1;TRINITY_DN69810_c2_g2;TRINITY_DN69810_c2_g1;TRINITY_DN71673_c1_g1;TRINITY_DN79388_c2_g2;TRINITY_DN73406_c0_g2(up);TRINITY_DN72050_c1_g2;TRINITY_DN79026_c0_g2(down);TRINITY_DN72893_c1_g2;TRINITY_DN66759_c1_g1;TRINITY_DN79587_c1_g1;TRINITY_DN76689_c1_g3;TRINITY_DN78755_c1_g1(down);TRINITY_DN62265_c2_g1;TRINITY_DN81176_c2_g3;TRINITY_DN78406_c1_g2	0.002775397
ko00030	Pentose phosphate pathway	34 /2030	66 /5870	TRINITY_DN71804_c0_g3;TRINITY_DN62681_c0_g1;TRINITY_DN54898_c0_g1;TRINITY_DN79679_c2_g1(down);TRINITY_DN60691_c1_g4;TRINITY_DN79031_c0_g1;TRINITY_DN53431_c0_g1;TRINITY_DN79505_c2_g1;TRINITY_DN76907_c1_g2;TRINITY_DN72609_c1_g1;TRINITY_DN77400_c0_g1;TRINITY_DN83109_c3_g1(up);TRINITY_DN68004_c2_g2(up);TRINITY_DN76548_c2_g1;TRINITY_DN62470_c1_g1;TRINITY_DN55506_c8_g1(up);TRINITY_DN72897_c0_g3;TRINITY_DN77599_c0_g3(down);TRINITY_DN69599_c0_g1(down);TRINITY_DN71838_c1_g1;TRINITY_DN58377_c0_g1;TRINITY_DN72814_c0_g1;TRINITY_DN77851_c0_g1;TRINITY_DN67443_c2_g3(down);TRINITY_DN65027_c0_g1;TRINITY_DN79817_c2_g1;TRINITY_DN52951_c0_g1;TRINITY_DN65162_c0_g2;TRINITY_DN79763_c2_g3;TRINITY_DN79505_c0_g1;TRINITY_DN56846_c0_g1;TRINITY_DN76269_c0_g3;TRINITY_DN77897_c1_g1(up);TRINITY_DN64613_c0_g3;TRINITY_DN80426_c1_g1;TRINITY_DN76548_c3_g1;TRINITY_DN75777_c1_g1(down);TRINITY_DN82322_c1_g2(up);TRINITY_DN75165_c0_g1;TRINITY_DN66656_c1_g2;TRINITY_DN80928_c3_g3;TRINITY_DN76269_c0_g2(up);TRINITY_DN74067_c0_g1;TRINITY_DN70349_c3_g3;TRINITY_DN77196_c1_g1;TRINITY_DN72897_c0_g1;TRINITY_DN70487_c4_g2(up);TRINITY_DN70270_c3_g2;TRINITY_DN77851_c3_g1;TRINITY_DN67108_c0_g1;TRINITY_DN78834_c1_g1(up);TRINITY_DN76355_c1_g1;TRINITY_DN53805_c0_g1(up);TRINITY_DN50455_c0_g1;TRINITY_DN79505_c1_g2;TRINITY_DN72893_c1_g2;TRINITY_DN70178_c0_g1;TRINITY_DN70178_c0_g2;TRINITY_DN65431_c0_g1;TRINITY_DN71185_c2_g1;TRINITY_DN78709_c2_g1;TRINITY_DN78117_c0_g1;TRINITY_DN62265_c2_g1;TRINITY_DN78117_c0_g2;TRINITY_DN65597_c0_g2(up);TRINITY_DN78406_c1_g2	0.003288887
ko00270	Cysteine and methionine metabolism	46 /2030	95 /5870	TRINITY_DN54709_c1_g2(down);TRINITY_DN59391_c0_g1;TRINITY_DN67581_c1_g1(down);TRINITY_DN65419_c0_g1;TRINITY_DN76865_c0_g1;TRINITY_DN65766_c1_g1;TRINITY_DN54088_c2_g3;TRINITY_DN68866_c1_g1;TRINITY_DN65129_c0_g1(up);TRINITY_DN66914_c1_g2;TRINITY_DN104807_c0_g1;TRINITY_DN73656_c0_g2(up);TRINITY_DN76795_c2_g1;TRINITY_DN74270_c0_g1;TRINITY_DN47297_c0_g1;TRINITY_DN65511_c3_g1(down);TRINITY_DN82039_c1_g1;TRINITY_DN77567_c0_g1;TRINITY_DN76295_c2_g1;TRINITY_DN77567_c0_g2;TRINITY_DN63307_c6_g2;TRINITY_DN45473_c0_g1;TRINITY_DN60773_c0_g1;TRINITY_DN79446_c1_g1(down);TRINITY_DN76575_c0_g2;TRINITY_DN66212_c2_g2;TRINITY_DN69453_c2_g5;TRINITY_DN52909_c0_g1(up);TRINITY_DN74408_c0_g1;TRINITY_DN66055_c1_g1;TRINITY_DN77144_c0_g2;TRINITY_DN54088_c2_g4;TRINITY_DN69088_c1_g1;TRINITY_DN76310_c2_g1;TRINITY_DN81747_c3_g2(down);TRINITY_DN53754_c0_g1(down);TRINITY_DN77907_c0_g4;TRINITY_DN82422_c1_g4;TRINITY_DN77907_c0_g3(down);TRINITY_DN69453_c2_g3(down);TRINITY_DN77489_c1_g1;TRINITY_DN81032_c1_g1;TRINITY_DN75105_c1_g6(up);TRINITY_DN67806_c0_g1(down);TRINITY_DN73401_c0_g1(down);TRINITY_DN71150_c2_g2(down);TRINITY_DN67254_c0_g3;TRINITY_DN79104_c1_g3;TRINITY_DN70392_c0_g2;TRINITY_DN63198_c1_g1(up);TRINITY_DN78403_c1_g2;TRINITY_DN79282_c1_g1;TRINITY_DN68314_c1_g1;TRINITY_DN67806_c0_g4(down);TRINITY_DN58360_c0_g1;TRINITY_DN71003_c0_g1;TRINITY_DN64536_c0_g1;TRINITY_DN62908_c0_g1;TRINITY_DN61856_c0_g3;TRINITY_DN71043_c0_g1;TRINITY_DN64472_c0_g1;TRINITY_DN75606_c1_g4;TRINITY_DN73382_c0_g1(up);TRINITY_DN73489_c0_g1;TRINITY_DN81756_c1_g1(down);TRINITY_DN78957_c0_g5;TRINITY_DN79104_c1_g4(up);TRINITY_DN75166_c1_g1;TRINITY_DN56931_c0_g1(down);TRINITY_DN82812_c7_g1;TRINITY_DN82812_c7_g2;TRINITY_DN76865_c1_g1;TRINITY_DN77445_c0_g1;TRINITY_DN69823_c1_g1;TRINITY_DN80196_c3_g1(down);TRINITY_DN78589_c1_g1(up);TRINITY_DN77434_c0_g1;TRINITY_DN79243_c2_g1;TRINITY_DN62966_c1_g2;TRINITY_DN81169_c2_g4;TRINITY_DN68685_c1_g1;TRINITY_DN82418_c2_g1;TRINITY_DN56107_c1_g1(down);TRINITY_DN78486_c2_g1;TRINITY_DN73019_c0_g1(down);TRINITY_DN62028_c1_g6;TRINITY_DN81612_c2_g2;TRINITY_DN75289_c0_g2;TRINITY_DN58699_c0_g1(up);TRINITY_DN66990_c1_g1(down);TRINITY_DN60770_c8_g1(down);TRINITY_DN48479_c0_g1;TRINITY_DN75458_c0_g2;TRINITY_DN70589_c0_g3(down);TRINITY_DN70986_c1_g1(up)	0.003491403

ko00941	**Flavonoid biosynthesis**	18/1644	36/5870	TRINITY_DN72283_c4_g2(up);TRINITY_DN75819_c0_g2;TRINITY_DN50284_c0_g1;TRINITY_DN67758_c0_g1;TRINITY_DN74752_c0_g1;TRINITY_DN70585_c1_g2(up);TRINITY_DN62989_c0_g2(down);TRINITY_DN65373_c0_g1(down);TRINITY_DN66813_c0_g4(up);TRINITY_DN74913_c1_g1;TRINITY_DN67504_c0_g2(down);TRINITY_DN35501_c0_g1;TRINITY_DN54818_c0_g1;TRINITY_DN63042_c0_g1;TRINITY_DN47547_c0_g1;TRINITY_DN60292_c0_g1;TRINITY_DN76638_c2_g1(down);TRINITY_DN55472_c1_g1(up);TRINITY_DN57504_c0_g1;TRINITY_DN56554_c5_g1(up);TRINITY_DN64844_c0_g3(up);TRINITY_DN71791_c0_g1;TRINITY_DN74752_c1_g1(up);TRINITY_DN82533_c3_g1;TRINITY_DN74863_c0_g1;TRINITY_DN57054_c0_g1(up);TRINITY_DN77352_c1_g2(up);TRINITY_DN71052_c0_g1(up);TRINITY_DN57937_c0_g1;TRINITY_DN82533_c1_g1;TRINITY_DN77213_c1_g2(up);TRINITY_DN77213_c1_g4;TRINITY_DN75177_c0_g3(up);TRINITY_DN75177_c0_g1(down);TRINITY_DN65847_c0_g2;TRINITY_DN71912_c1_g1(down)	0.004129326
ko01230	Biosynthesis of amino acids	113/2030	267/5870	TRINITY_DN54709_c1_g2(down);TRINITY_DN76475_c1_g2(up);TRINITY_DN71804_c0_g3;TRINITY_DN59391_c0_g1;TRINITY_DN75572_c0_g1(up);TRINITY_DN74076_c1_g1(down);TRINITY_DN56575_c1_g1(up);TRINITY_DN79540_c1_g1;TRINITY_DN61342_c1_g2(down);TRINITY_DN78059_c1_g3(up);TRINITY_DN66755_c0_g1;TRINITY_DN76548_c2_g1;TRINITY_DN75582_c0_g1(up);TRINITY_DN83109_c3_g1(up);TRINITY_DN65419_c0_g1;TRINITY_DN71989_c0_g4;TRINITY_DN78958_c2_g1;TRINITY_DN70861_c2_g1;TRINITY_DN80257_c2_g1(down);TRINITY_DN68576_c0_g1;TRINITY_DN72242_c0_g1;TRINITY_DN66894_c0_g1;TRINITY_DN67359_c0_g1;TRINITY_DN80010_c1_g1;TRINITY_DN77326_c1_g1(up);TRINITY_DN77907_c0_g2(down);TRINITY_DN73489_c0_g1;TRINITY_DN69762_c1_g2;TRINITY_DN64837_c1_g2;TRINITY_DN79463_c0_g2;TRINITY_DN79463_c0_g3;TRINITY_DN80978_c1_g2;TRINITY_DN75075_c0_g3(down);TRINITY_DN78576_c0_g2;TRINITY_DN72532_c2_g1;TRINITY_DN78109_c0_g1;TRINITY_DN68189_c0_g3(up);TRINITY_DN75517_c0_g1;TRINITY_DN79052_c2_g1(up);TRINITY_DN80650_c1_g2;TRINITY_DN74721_c0_g1;TRINITY_DN79719_c3_g1(down);TRINITY_DN77407_c3_g2(up);TRINITY_DN76269_c0_g2(up);TRINITY_DN79994_c1_g1;TRINITY_DN74067_c0_g1;TRINITY_DN40338_c0_g1;TRINITY_DN79520_c0_g1;TRINITY_DN69291_c2_g2;TRINITY_DN73944_c1_g1;TRINITY_DN76201_c0_g1(down);TRINITY_DN60163_c1_g1(down);TRINITY_DN70230_c0_g1;TRINITY_DN76699_c2_g2;TRINITY_DN72075_c1_g2;TRINITY_DN65511_c3_g1(down);TRINITY_DN53805_c0_g1(up);TRINITY_DN79267_c3_g1;TRINITY_DN59441_c0_g1;TRINITY_DN63307_c6_g2;TRINITY_DN60864_c1_g1(down);TRINITY_DN82039_c1_g1;TRINITY_DN79901_c2_g1;TRINITY_DN74510_c3_g2;TRINITY_DN77993_c5_g1;TRINITY_DN76744_c1_g3;TRINITY_DN78201_c1_g2;TRINITY_DN78201_c1_g3;TRINITY_DN62470_c1_g1;TRINITY_DN62681_c0_g1;TRINITY_DN67297_c1_g3;TRINITY_DN67297_c1_g2;TRINITY_DN66212_c2_g2;TRINITY_DN81216_c3_g2(down);TRINITY_DN71834_c1_g2(down);TRINITY_DN69453_c2_g5;TRINITY_DN77851_c0_g1;TRINITY_DN82434_c2_g2(down);TRINITY_DN79791_c1_g1;TRINITY_DN70702_c0_g2;TRINITY_DN62932_c0_g1;TRINITY_DN78933_c0_g1;TRINITY_DN67581_c1_g1(down);TRINITY_DN74786_c2_g3;TRINITY_DN68004_c2_g2(up);TRINITY_DN52789_c0_g1;TRINITY_DN61153_c0_g1(down);TRINITY_DN79240_c1_g1;TRINITY_DN73252_c1_g1;TRINITY_DN69481_c5_g5;TRINITY_DN56813_c0_g1(down);TRINITY_DN75258_c1_g1;TRINITY_DN74729_c0_g1;TRINITY_DN69481_c5_g1(down);TRINITY_DN49467_c0_g1;TRINITY_DN52168_c0_g2;TRINITY_DN65358_c1_g3(up);TRINITY_DN76548_c3_g1;TRINITY_DN72759_c1_g1(down);TRINITY_DN66810_c1_g3;TRINITY_DN66810_c1_g2;TRINITY_DN78994_c1_g1(down);TRINITY_DN71378_c0_g1;TRINITY_DN76269_c0_g3;TRINITY_DN70347_c0_g1(up);TRINITY_DN65139_c0_g1;TRINITY_DN55843_c6_g3;TRINITY_DN79026_c0_g2(down);TRINITY_DN54135_c2_g2;TRINITY_DN69088_c1_g1;TRINITY_DN76310_c2_g1;TRINITY_DN81747_c3_g2(down);TRINITY_DN56931_c0_g1(down);TRINITY_DN78639_c1_g2(down);TRINITY_DN72296_c1_g4;TRINITY_DN75611_c1_g2(up);TRINITY_DN81830_c2_g3;TRINITY_DN69453_c2_g3(down);TRINITY_DN52809_c1_g1;TRINITY_DN77600_c1_g1;TRINITY_DN74270_c0_g1;TRINITY_DN77716_c1_g1(down);TRINITY_DN80035_c2_g1;TRINITY_DN77547_c0_g1;TRINITY_DN77489_c1_g1;TRINITY_DN67889_c2_g4;TRINITY_DN75460_c3_g1;TRINITY_DN78059_c1_g4(up);TRINITY_DN75105_c1_g6(up);TRINITY_DN54505_c1_g1;TRINITY_DN74936_c0_g1(down);TRINITY_DN75414_c0_g2;TRINITY_DN75335_c0_g4(down);TRINITY_DN71150_c2_g2(down);TRINITY_DN74955_c0_g3;TRINITY_DN76040_c0_g2;TRINITY_DN64576_c5_g1;TRINITY_DN76793_c4_g1;TRINITY_DN67443_c2_g3(down);TRINITY_DN50716_c0_g1(up);TRINITY_DN79104_c1_g3;TRINITY_DN50589_c0_g2;TRINITY_DN50877_c1_g1;TRINITY_DN63596_c0_g2;TRINITY_DN70392_c0_g2;TRINITY_DN55506_c8_g1(up);TRINITY_DN61264_c2_g5;TRINITY_DN67329_c0_g1;TRINITY_DN74510_c2_g1;TRINITY_DN75559_c2_g1(down);TRINITY_DN67329_c0_g2;TRINITY_DN73585_c1_g1;TRINITY_DN83195_c11_g1(down);TRINITY_DN82733_c3_g1(down);TRINITY_DN52951_c0_g1;TRINITY_DN79107_c1_g1;TRINITY_DN68314_c1_g1;TRINITY_DN55276_c0_g1;TRINITY_DN79181_c1_g3;TRINITY_DN77891_c0_g1;TRINITY_DN78834_c1_g1(up);TRINITY_DN64588_c0_g2(down);TRINITY_DN58360_c0_g1;TRINITY_DN64613_c0_g3;TRINITY_DN80426_c1_g1;TRINITY_DN64823_c1_g1;TRINITY_DN56378_c0_g1;TRINITY_DN77031_c2_g2;TRINITY_DN83048_c2_g1(down);TRINITY_DN71003_c0_g1;TRINITY_DN78109_c1_g1;TRINITY_DN82322_c1_g2(up);TRINITY_DN61856_c0_g3;TRINITY_DN66656_c1_g2;TRINITY_DN77955_c1_g1;TRINITY_DN61774_c2_g1;TRINITY_DN55308_c0_g2(up);TRINITY_DN77407_c3_g1(up);TRINITY_DN64472_c0_g1;TRINITY_DN75727_c0_g1;TRINITY_DN80328_c7_g2;TRINITY_DN80328_c7_g1(up);TRINITY_DN77851_c3_g1;TRINITY_DN58504_c4_g2;TRINITY_DN56846_c0_g1;TRINITY_DN60071_c3_g1;TRINITY_DN79858_c1_g1(up);TRINITY_DN78957_c0_g5;TRINITY_DN79104_c1_g4(up);TRINITY_DN72345_c0_g1;TRINITY_DN60680_c2_g2;TRINITY_DN78703_c0_g3(down);TRINITY_DN80874_c4_g4;TRINITY_DN67899_c3_g1;TRINITY_DN78639_c1_g5(down);TRINITY_DN50809_c0_g1(down);TRINITY_DN59312_c0_g1;TRINITY_DN68562_c0_g2;TRINITY_DN83222_c6_g1;TRINITY_DN67499_c5_g1;TRINITY_DN78427_c3_g3;TRINITY_DN79031_c0_g1;TRINITY_DN78276_c2_g3;TRINITY_DN78276_c2_g2;TRINITY_DN77681_c2_g2;TRINITY_DN54505_c1_g2;TRINITY_DN67049_c0_g1;TRINITY_DN77681_c2_g5;TRINITY_DN78276_c2_g4;TRINITY_DN57160_c0_g1;TRINITY_DN73877_c0_g2;TRINITY_DN60374_c0_g3;TRINITY_DN75745_c1_g1;TRINITY_DN78059_c1_g1(up);TRINITY_DN60770_c8_g1(down);TRINITY_DN80196_c3_g1(down);TRINITY_DN79600_c3_g1;TRINITY_DN81423_c2_g1;TRINITY_DN57505_c0_g1(down);TRINITY_DN64893_c1_g1(down);TRINITY_DN64639_c0_g1;TRINITY_DN65027_c0_g1;TRINITY_DN57169_c0_g1(down);TRINITY_DN67937_c4_g3;TRINITY_DN82588_c2_g1;TRINITY_DN75325_c1_g6;TRINITY_DN67706_c0_g2;TRINITY_DN81169_c2_g4;TRINITY_DN64536_c0_g1;TRINITY_DN72071_c0_g1;TRINITY_DN79434_c2_g1;TRINITY_DN76438_c0_g1;TRINITY_DN62908_c0_g1;TRINITY_DN66055_c1_g1;TRINITY_DN79424_c1_g1(up);TRINITY_DN79165_c2_g1(down);TRINITY_DN75165_c0_g1;TRINITY_DN80119_c1_g1(down);TRINITY_DN76699_c2_g1;TRINITY_DN51840_c0_g2(up);TRINITY_DN79388_c2_g2;TRINITY_DN77153_c1_g1;TRINITY_DN47487_c0_g1;TRINITY_DN70986_c1_g1(up);TRINITY_DN69414_c0_g1;TRINITY_DN73401_c0_g1(down);TRINITY_DN75289_c0_g2;TRINITY_DN74408_c0_g1;TRINITY_DN75335_c0_g1(down);TRINITY_DN54580_c0_g1(down);TRINITY_DN66326_c0_g1;TRINITY_DN54852_c0_g3;TRINITY_DN72893_c1_g2;TRINITY_DN66759_c1_g1;TRINITY_DN56180_c0_g1(up);TRINITY_DN64726_c3_g1;TRINITY_DN71185_c2_g1;TRINITY_DN79994_c2_g1;TRINITY_DN70349_c3_g3;TRINITY_DN75606_c1_g4;TRINITY_DN75582_c0_g2;TRINITY_DN62685_c2_g1;TRINITY_DN70589_c0_g3(down);TRINITY_DN78620_c0_g1(down);TRINITY_DN78406_c1_g2	0.004343019
ko00052	Galactose metabolism	24/2030	45/5870	TRINITY_DN72699_c1_g2(down);TRINITY_DN73291_c1_g1;TRINITY_DN79679_c2_g1(down);TRINITY_DN73603_c2_g3;TRINITY_DN62209_c1_g1;TRINITY_DN74103_c1_g1;TRINITY_DN78573_c0_g2;TRINITY_DN78086_c1_g3;TRINITY_DN77400_c0_g1;TRINITY_DN68004_c2_g2(up);TRINITY_DN82958_c3_g2;TRINITY_DN75644_c1_g3(down);TRINITY_DN65671_c0_g1;TRINITY_DN74965_c0_g1;TRINITY_DN62209_c2_g1(up);TRINITY_DN67964_c1_g1;TRINITY_DN75539_c2_g1(down);TRINITY_DN79587_c1_g1;TRINITY_DN72841_c1_g2(down);TRINITY_DN82872_c8_g3;TRINITY_DN76195_c0_g1;TRINITY_DN78834_c1_g1(up);TRINITY_DN76269_c0_g3;TRINITY_DN79380_c1_g1(up);TRINITY_DN81577_c7_g1;TRINITY_DN76327_c1_g4;TRINITY_DN77505_c0_g1(up);TRINITY_DN81647_c1_g3(up);TRINITY_DN82322_c1_g2(up);TRINITY_DN71673_c1_g1;TRINITY_DN80928_c3_g3;TRINITY_DN76269_c0_g2(up);TRINITY_DN76360_c0_g2;TRINITY_DN76360_c0_g1;TRINITY_DN65875_c1_g1;TRINITY_DN68483_c7_g1;TRINITY_DN77790_c2_g1(down);TRINITY_DN77232_c0_g1;TRINITY_DN62774_c1_g1(up);TRINITY_DN77106_c1_g2(down);TRINITY_DN69392_c2_g1;TRINITY_DN50382_c0_g2(down);TRINITY_DN78755_c1_g1(down);TRINITY_DN82998_c8_g2;TRINITY_DN67269_c0_g1	0.007309034
ko00410	beta-Alanine metabolism	25/2030	48/5870	TRINITY_DN72325_c0_g1(down);TRINITY_DN56347_c4_g5;TRINITY_DN80219_c3_g1;TRINITY_DN78031_c0_g3;TRINITY_DN79467_c1_g2(down);TRINITY_DN80208_c5_g1(down);TRINITY_DN79840_c3_g1(down);TRINITY_DN61815_c0_g2(up);TRINITY_DN71321_c0_g2;TRINITY_DN79467_c1_g1(up);TRINITY_DN70374_c0_g3(up);TRINITY_DN76959_c0_g2;TRINITY_DN74175_c1_g1;TRINITY_DN71719_c2_g1;TRINITY_DN78031_c0_g4(down);TRINITY_DN72624_c0_g1;TRINITY_DN67224_c1_g2;TRINITY_DN80144_c3_g1;TRINITY_DN78031_c0_g1(up);TRINITY_DN82118_c4_g1;TRINITY_DN79243_c2_g1;TRINITY_DN69956_c4_g2;TRINITY_DN69956_c4_g1;TRINITY_DN75756_c0_g1(up);TRINITY_DN66919_c0_g1;TRINITY_DN74870_c0_g1;TRINITY_DN74814_c0_g2;TRINITY_DN50774_c0_g1(down);TRINITY_DN83085_c3_g1;TRINITY_DN73656_c0_g2(up);TRINITY_DN74175_c1_g2(down);TRINITY_DN67407_c0_g1;TRINITY_DN81296_c4_g1;TRINITY_DN69709_c1_g1(down);TRINITY_DN55923_c0_g1(down);TRINITY_DN83085_c3_g2;TRINITY_DN65118_c0_g1;TRINITY_DN78051_c6_g3;TRINITY_DN76864_c0_g2(down);TRINITY_DN98023_c0_g1;TRINITY_DN74814_c0_g3;TRINITY_DN60378_c0_g	0.009221968

				1;TRINITY_DN78253_c2_g3;TRINITY_DN72951_c0_g4(up);TRINITY_DN74842_c1_g4;TRINITY_DN55451_c0_g1;TRINITY_DN74842_c1_g1;TRINITY_DN78253_c2_g1(up)	
ko00400	Phenylalanine, tyrosine and tryptophan biosynthesis	29/2030	58/5870	TRINITY_DN74955_c0_g3;TRINITY_DN63596_c0_g2;TRINITY_DN67297_c1_g3;TRINITY_DN83222_c6_g1;TRINITY_DN75572_c0_g1(up);TRINITY_DN50716_c0_g1(up);TRINITY_DN74076_c1_g1(down);TRINITY_DN56575_c1_g1(up);TRINITY_DN50589_c0_g2;TRINITY_DN78059_c1_g3(up);TRINITY_DN57160_c0_g1;TRINITY_DN75582_c0_g1(up);TRINITY_DN75745_c1_g1;TRINITY_DN73944_c1_g1;TRINITY_DN79240_c1_g1;TRINITY_DN70861_c2_g1;TRINITY_DN68576_c0_g1;TRINITY_DN73252_c1_g1;TRINITY_DN74408_c0_g1;TRINITY_DN60770_c8_g1(down);TRINITY_DN56180_c0_g1(up);TRINITY_DN69481_c5_g1(down);TRINITY_DN67297_c1_g2;TRINITY_DN77326_c1_g1(up);TRINITY_DN65358_c1_g3(up);TRINITY_DN66810_c1_g3;TRINITY_DN55276_c0_g1;TRINITY_DN79181_c1_g3;TRINITY_DN75325_c1_g6;TRINITY_DN70347_c0_g1(up);TRINITY_DN66810_c1_g2;TRINITY_DN79434_c2_g1;TRINITY_DN76438_c0_g1;TRINITY_DN70702_c0_g2;TRINITY_DN69088_c1_g1;TRINITY_DN77600_c1_g1;TRINITY_DN77407_c3_g2(up);TRINITY_DN75258_c1_g1;TRINITY_DN80119_c1_g1(down);TRINITY_DN79994_c1_g1;TRINITY_DN77407_c3_g1(up);TRINITY_DN64472_c0_g1;TRINITY_DN79520_c0_g1;TRINITY_DN75289_c0_g2;TRINITY_DN78059_c1_g1(up);TRINITY_DN74270_c0_g1;TRINITY_DN72242_c0_g1;TRINITY_DN80328_c7_g2;TRINITY_DN80328_c7_g1(up);TRINITY_DN52809_c1_g1;TRINITY_DN73489_c0_g1;TRINITY_DN69481_c5_g5;TRINITY_DN78059_c1_g4(up);TRINITY_DN51840_c0_g2(up);TRINITY_DN75582_c0_g2;TRINITY_DN75517_c0_g1;TRINITY_DN79994_c2_g1;TRINITY_DN71989_c0_g4	0.010805364
ko00710	Carbon fixation in photosynthetic organisms	36/2030	75/5870	TRINITY_DN79600_c3_g1;TRINITY_DN62470_c1_g1;TRINITY_DN62681_c0_g1;TRINITY_DN72893_c1_g2;TRINITY_DN65162_c0_g2;TRINITY_DN79031_c0_g1;TRINITY_DN67313_c0_g1(down);TRINITY_DN78276_c2_g3;TRINITY_DN52045_c0_g2(down);TRINITY_DN108510_c0_g1;TRINITY_DN77993_c5_g1;TRINITY_DN83109_c3_g1(up);TRINITY_DN78276_c2_g4;TRINITY_DN76040_c0_g2;TRINITY_DN78782_c2_g1(up);TRINITY_DN52789_c0_g1;TRINITY_DN82649_c1_g1;TRINITY_DN61264_c2_g5;TRINITY_DN71838_c1_g1;TRINITY_DN55506_c8_g1(up);TRINITY_DN70284_c0_g1(down);TRINITY_DN68313_c1_g2;TRINITY_DN31309_c0_g1;TRINITY_DN80010_c1_g1;TRINITY_DN73271_c0_g2;TRINITY_DN65027_c0_g1;TRINITY_DN79817_c2_g1;TRINITY_DN72896_c0_g2(down);TRINITY_DN77044_c5_g2(down);TRINITY_DN104807_c0_g1;TRINITY_DN66911_c1_g1;TRINITY_DN56846_c0_g1;TRINITY_DN81896_c4_g4;TRINITY_DN76773_c1_g2;TRINITY_DN76773_c1_g3;TRINITY_DN64613_c0_g3;TRINITY_DN80426_c1_g1;TRINITY_DN80629_c3_g1(up);TRINITY_DN83048_c2_g1(down);TRINITY_DN74721_c0_g1;TRINITY_DN50551_c0_g1;TRINITY_DN53754_c0_g1(down);TRINITY_DN75165_c0_g1; TRINITY_DN66656_c1_g2;TRINITY_DN74067_c0_g1;TRINITY_DN64472_c0_g1;TRINITY_DN73019_c0_g1(down);TRINITY_DN58641_c2_g1;TRINITY_DN82649_c2_g2(down);TRINITY_DN62028_c1_g6;TRINITY_DN71378_c0_g1;TRINITY_DN74270_c0_g1;TRINITY_DN77891_c0_g1;TRINITY_DN74148_c0_g1;TRINITY_DN80088_c1_g1;TRINITY_DN78276_c2_g2;TRINITY_DN76712_c0_g1;TRINITY_DN79026_c0_g2(down);TRINITY_DN74338_c1_g1(down);TRINITY_DN76355_c1_g1;TRINITY_DN53805_c0_g1(up);TRINITY_DN77567_c0_g1;TRINITY_DN71185_c2_g1;TRINITY_DN69451_c0_g2;TRINITY_DN77567_c0_g2;TRINITY_DN66990_c1_g1(down);TRINITY_DN60770_c8_g1(down);TRINITY_DN75166_c1_g1;TRINITY_DN68189_c0_g3(up);TRINITY_DN78589_c1_g1(up);TRINITY_DN79901_c2_g1;TRINITY_DN62265_c2_g1;TRINITY_DN64356_c1_g3;TRINITY_DN62026_c1_g2(down);TRINITY_DN78406_c1_g2	0.010845865
ko04152	AMPK signaling pathway	44/2030	96/5870	TRINITY_DN72618_c1_g2;TRINITY_DN51249_c0_g1;TRINITY_DN71997_c1_g1;TRINITY_DN75928_c0_g2(down);TRINITY_DN82006_c5_g1;TRINITY_DN72465_c1_g1;TRINITY_DN48807_c0_g1;TRINITY_DN72624_c0_g1;TRINITY_DN78443_c1_g1(up);TRINITY_DN75356_c0_g2;TRINITY_DN67066_c0_g3(down);TRINITY_DN76269_c0_g2(up);TRINITY_DN73672_c0_g1;TRINITY_DN81316_c1_g1(down);TRINITY_DN69546_c2_g5(down);TRINITY_DN74238_c0_g1(up);TRINITY_DN67210_c0_g1(down);TRINITY_DN78536_c1_g4(up);TRINITY_DN51432_c0_g1;TRINITY_DN69859_c1_g1;TRINITY_DN83050_c1_g1;TRINITY_DN57117_c2_g1;TRINITY_DN66593_c1_g2;TRINITY_DN82508_c4_g1;TRINITY_DN71838_c1_g1;TRINITY_DN80356_c4_g1(up);TRINITY_DN62285_c0_g1;TRINITY_DN46512_c0_g2;TRINITY_DN68004_c2_g2(up);TRINITY_DN60391_c3_g2;TRINITY_DN55014_c0_g2;TRINITY_DN55498_c0_g1;TRINITY_DN67728_c0_g3;TRINITY_DN82814_c9_g2;TRINITY_DN81276_c3_g2(down);TRINITY_DN79817_c2_g1;TRINITY_DN75956_c1_g1(down);TRINITY_DN65952_c0_g1(up);TRINITY_DN76269_c0_g3;TRINITY_DN61651_c0_g1;TRINITY_DN82185_c3_g4;TRINITY_DN70639_c1_g1;TRINITY_DN77678_c0_g1(down);TRINITY_DN65953_c3_g1(down);TRINITY_DN67066_c1_g1(down);TRINITY_DN56625_c4_g1;TRINITY_DN66961_c0_g1;TRINITY_DN83080_c5_g2(down);TRINITY_DN77472_c1_g2(up);TRINITY_DN82326_c2_g1;TRINITY_DN82326_c2_g2;TRINITY_DN63398_c2_g1;TRINITY_DN73774_c1_g1;TRINITY_DN57964_c0_g1;TRINITY_DN82322_c1_g2(up);TRINITY_DN78834_c1_g1(up);TRINITY_DN65752_c4_g1;TRINITY_DN78857_c1_g1;TRINITY_DN81092_c1_g1;TRINITY_DN81342_c2_g1(down);TRINITY_DN83043_c9_g1;TRINITY_DN51506_c0_g1;TRINITY_DN60147_c0_g5(up);TRINITY_DN78623_c1_g1;TRINITY_DN58842_c0_g2;TRINITY_DN71143_c0_g1;TRINITY_DN76355_c1_g1;TRINITY_DN71143_c0_g2;TRINITY_DN65221_c0_g2(up);TRINITY_DN69546_c2_g4;TRINITY_DN50176_c0_g1;TRINITY_DN75960_c2_g1;TRINITY_DN65162_c0_g2;TRINITY_DN62285_c0_g3;TRINITY_DN57778_c0_g1;TRINITY_DN77561_c1_g4;TRINITY_DN75749_c0_g2(down);TRINITY_DN79341_c3_g1;TRINITY_DN70607_c0_g2(down);TRINITY_DN82016_c4_g6;TRINITY_DN45980_c0_g1;TRINITY_DN62645_c0_g1;TRINITY_DN83080_c4_g1;TRINITY_DN80229_c2_g2;TRINITY_DN80889_c1_g1(down);TRINITY_DN82326_c1_g2;TRINITY_DN57117_c4_g1;TRINITY_DN79722_c2_g1;TRINITY_DN79503_c0_g1(down);TRINITY_DN81941_c2_g1(down);TRINITY_DN47296_c0_g1;TRINITY_DN65788_c4_g4;TRINITY_DN62265_c2_g1;TRINITY_DN58668_c0_g1;TRINITY_DN63227_c1_g1(up);TRINITY_DN82478_c1_g3(down)	0.014075366
ko04145	Phagosome	43/2030	95/5870	TRINITY_DN77495_c0_g2;TRINITY_DN74452_c2_g4;TRINITY_DN52358_c0_g3(down);TRINITY_DN64534_c3_g3(up);TRINITY_DN69497_c0_g3;TRINITY_DN65297_c0_g2(down);TRINITY_DN82931_c1_g2(down);TRINITY_DN82941_c2_g2;TRINITY_DN82941_c2_g1;TRINITY_DN62390_c1_g4;TRINITY_DN76352_c3_g1;TRINITY_DN67309_c1_g2(down);TRINITY_DN71141_c0_g1;TRINITY_DN73850_c0_g2(down);TRINITY_DN51219_c0_g1;TRINITY_DN57411_c0_g1(down);TRINITY_DN72805_c4_g1;TRINITY_DN69264_c1_g2;TRINITY_DN47468_c0_g1;TRINITY_DN56188_c0_g2;TRINITY_DN52037_c0_g2;TRINITY_DN72371_c0_g3(up);TRINITY_DN63381_c0_g2(down);TRINITY_DN81916_c4_g3;TRINITY_DN69264_c1_g1(down);TRINITY_DN79564_c0_g2(down);TRINITY_DN76216_c1_g3(up);TRINITY_DN74941_c0_g1;TRINITY_DN92183_c0_g1;TRINITY_DN69401_c0_g1(up);TRINITY_DN75615_c1_g3;TRINITY_DN67633_c5_g2;TRINITY_DN70851_c2_g5;TRINITY_DN82187_c1_g4;TRINITY_DN58779_c0_g1;TRINITY_DN82931_c2_g1;TRINITY_DN80204_c0_g1(down);TRINITY_DN77495_c1_g2;TRINITY_DN54754_c0_g1;TRINITY_DN70977_c1_g1;TRINITY_DN79296_c1_g1;TRINITY_DN79678_c3_g1(up);TRINITY_DN79538_c1_g2;TRINITY_DN79538_c1_g3;TRINITY_DN73364_c1_g1;TRINITY_DN63784_c0_g1(down);TRINITY_DN67469_c0_g2;TRINITY_DN67593_c5_g6;TRINITY_DN65805_c1_g5;TRINITY_DN65805_c1_g8;TRINITY_DN66133_c0_g2(down);TRINITY_DN81957_c5_g1;TRINITY_DN55244_c0_g5(up);TRINITY_DN78340_c0_g3(down);TRINITY_DN82790_c0_g2;TRINITY_DN56032_c1_g1;TRINITY_DN82655_c1_g1;TRINITY_DN78660_c1_g1;TRINITY_DN47918_c0_g1;TRINITY_DN82655_c0_g1;TRINITY_DN64491_c0_g1;TRINITY_DN78340_c0_g4;TRINITY_DN75615_c1_g2;TRINITY_DN54793_c0_g1;TRINITY_DN57583_c1_g1;TRINITY_DN68931_c0_g1;TRINITY_DN82908_c3_g1;TRINITY_DN78372_c4_g2;TRINITY_DN70851_c2_g1;TRINITY_DN79006_c0_g4;TRINITY_DN48235_c0_g2;TRINITY_DN79538_c2_g2(down);TRINITY_DN49120_c1_g1;TRINITY_DN78211_c5_g2;TRINITY_DN74443_c0_g1;TRINITY_DN73142_c1_g2;TRINITY_DN78190_c1_g1;TRINITY_DN62812_c0_g4(up);TRINITY_DN71513_c1_g6;TRINITY_DN78317_c2_g1;TRINITY_DN69226_c1_g1;TRINITY_DN68801_c0_g2;TRINITY_DN65331_c0_g1;TRINITY_DN58936_c0_g1;TRINITY_DN73365_c3_g1;TRINITY_DN80362_c1_g1;TRINITY_DN82790_c1_g1;TRINITY_DN56120_c0_g1(down);TRINITY_DN65597_c1_g1;TRINITY_DN64717_c0_g2(up);TRINITY_DN73072_c0_g6(up);TRINITY_DN56120_c0_g2;TRINITY_DN82083_c2_g1(down);TRINITY_DN81755_c2_g1;TRINITY_DN81682_c1_g1(down)	0.01928776
ko00330	Arginine and proline metabolism	29/2030	62/5870	TRINITY_DN70683_c0_g2(up);TRINITY_DN69974_c1_g1;TRINITY_DN68841_c0_g1;TRINITY_DN79363_c1_g1(up);TRINITY_DN78343_c2_g1;TRINITY_DN78031_c0_g3;TRINITY_DN55923_c0_g1(down);TRINITY_DN79840_c3_g1(down);TRINITY_DN74814_c0_g2;TRINITY_DN78253_c2_g1(up);TRINITY_DN69344_c0_g1;TRINITY_DN52909_c0_g1(up);TRINITY_DN74510_c2_g1;TRINITY_DN72781_c0_g1;TRINITY_DN47487_c0_g1;TRINITY_DN60770_c8_g1(down);TRINITY_DN76582_c0_g2(down);TRINITY_DN57002_c1_g1;TRINITY_DN79363_c1_g3(up);TRINITY_DN72840_c1_g1;TRINITY_DN80144_c3_g1;TRINITY_DN74851_c1_g1;TRINITY_DN78031_c0_g1(up);TRINITY_DN82588_c2_g1;TRINITY_DN78576_c0_g2;TRINITY_DN79243_c2_g1;TRINITY_DN80317_c0_g1(up);TRINITY_DN77634_c1_g1(up);TRINITY_DN74922_c2_g1(up);TRINITY_DN69956_c4_g1;TRINITY_DN61399_c4_g1;TRINITY_DN74814_c0_g3;TRINITY_DN105246_c0_g1;TRINITY_DN52647_c0_g1;TRINITY_DN76730_c4_g2;TRINITY_DN80317_c2_g1;TRINITY_DN83085_c3_g2;TRINITY_DN78486_c2_g1;TRINITY_DN50774_c0_g1(down);TRINITY_DN64472_c0_g1;TRINITY_DN73656_c0_g2(up);TRINITY_DN79943_c1_g1(down);TRINITY_DN74270_c0_g1;TRINITY_DN55451_c0_g1;TRINITY_DN79959_c2_g2;TRINITY_DN73489_c0_g1;TRINITY_DN75727_c0_g1;TRINITY_DN69956_c4_g2;TRINITY_DN98023_c0_g1;TRINITY_DN78031_c0_g4(down);TRINITY_DN59837_c0_g1;TRI	0.030886124

				NITY_DN78253_c2_g3;TRINITY_DN72951_c0_g4(up);TRINITY_DN85704_c0_g1;TRINITY_DN83085_c3_g1;TRINITY_DN50542_c0_g1(up);TRINITY_DN71160_c0_g2;TRINITY_DN82112_c1_g3;TRINITY_DN59627_c2_g1;TRINITY_DN60773_c0_g1;TRINITY_DN77634_c2_g2(up);TRINITY_DN74510_c3_g2	
ko00906	Carotenoid biosynthesis	19 /2030	38 /5870	TRINITY_DN47781_c0_g1(up);TRINITY_DN79284_c0_g1;TRINITY_DN80577_c1_g1(up);TRINITY_DN81410_c5_g1;TRINITY_DN49920_c0_g1;TRINITY_DN62755_c0_g2;TRINITY_DN73695_c1_g1;TRINITY_DN73695_c1_g4;TRINITY_DN80798_c3_g1;TRINITY_DN74450_c0_g1(down);TRINITY_DN79217_c2_g1;TRINITY_DN62061_c2_g1;TRINITY_DN64392_c0_g1(up);TRINITY_DN70793_c0_g1(down);TRINITY_DN73256_c1_g2;TRINITY_DN75000_c0_g3;TRINITY_DN68413_c5_g1;TRINITY_DN70474_c1_g2;TRINITY_DN78178_c1_g1;TRINITY_DN75627_c0_g1(up);TRINITY_DN77665_c0_g1(down);TRINITY_DN73256_c0_g1;TRINITY_DN64150_c1_g1;TRINITY_DN80244_c2_g1(up);TRINITY_DN74450_c0_g2(down);TRINITY_DN74728_c2_g1(up);TRINITY_DN71848_c0_g3;TRINITY_DN71848_c0_g2;TRINITY_DN82995_c11_g4(up);TRINITY_DN64584_c1_g1;TRINITY_DN66338_c0_g1(up);TRINITY_DN80076_c1_g1;TRINITY_DN52507_c0_g1;TRINITY_DN78934_c0_g1;TRINITY_DN77665_c1_g1(down);TRINITY_DN106073_c0_g1;TRINITY_DN80658_c1_g2(up);TRINITY_DN51031_c0_g1	0.035611237
ko03018	RNA degradation	65 /2030	156 /5870	TRINITY_DN77823_c1_g1;TRINITY_DN82987_c0_g1(down);TRINITY_DN65176_c1_g4;TRINITY_DN77363_c0_g4;TRINITY_DN77363_c0_g1;TRINITY_DN70518_c0_g2(up);TRINITY_DN63762_c0_g1;TRINITY_DN71688_c2_g1;TRINITY_DN71144_c0_g2;TRINITY_DN69989_c4_g3(down);TRINITY_DN69995_c8_g1(down);TRINITY_DN68186_c0_g1(down);TRINITY_DN81533_c2_g1;TRINITY_DN69681_c1_g1;TRINITY_DN64837_c1_g2;TRINITY_DN73004_c1_g1;TRINITY_DN79757_c1_g1;TRINITY_DN68436_c0_g2;TRINITY_DN60959_c0_g1(down);TRINITY_DN79361_c0_g1(down);TRINITY_DN76269_c0_g2(up);TRINITY_DN82055_c3_g1;TRINITY_DN83223_c5_g1(down);TRINITY_DN68109_c0_g3;TRINITY_DN78061_c0_g1(down);TRINITY_DN76379_c1_g3;TRINITY_DN65764_c0_g1;TRINITY_DN79757_c1_g2(down);TRINITY_DN79267_c3_g1;TRINITY_DN50727_c0_g1;TRINITY_DN52272_c0_g1;TRINITY_DN78988_c1_g1(down);TRINITY_DN82275_c1_g1(down);TRINITY_DN70256_c1_g2;TRINITY_DN78061_c0_g5;TRINITY_DN71911_c0_g1;TRINITY_DN73188_c0_g4;TRINITY_DN82596_c2_g1(down);TRINITY_DN69958_c1_g1;TRINITY_DN73188_c0_g1(down);TRINITY_DN81765_c3_g1;TRINITY_DN73573_c3_g2(up);TRINITY_DN64140_c0_g4;TRINITY_DN56694_c2_g3;TRINITY_DN62148_c0_g3;TRINITY_DN83176_c3_g4(down);TRINITY_DN79792_c1_g1;TRINITY_DN76621_c0_g1;TRINITY_DN60456_c0_g1;TRINITY_DN55906_c4_g1(down);TRINITY_DN58630_c2_g3;TRINITY_DN81314_c2_g1(down);TRINITY_DN48311_c0_g2;TRINITY_DN72504_c1_g1;TRINITY_DN73199_c1_g1;TRINITY_DN82275_c2_g5;TRINITY_DN83034_c3_g1;TRINITY_DN76269_c0_g3;TRINITY_DN55843_c6_g2;TRINITY_DN52995_c1_g2;TRINITY_DN49155_c0_g1;TRINITY_DN65020_c0_g1(down);TRINITY_DN83034_c2_g2;TRINITY_DN50450_c0_g1(down);TRINITY_DN76838_c0_g4(down);TRINITY_DN82614_c1_g3(down);TRINITY_DN56884_c1_g3;TRINITY_DN72065_c0_g1(down);TRINITY_DN62666_c1_g2;TRINITY_DN53828_c3_g1;TRINITY_DN82819_c4_g1;TRINITY_DN68461_c0_g1;TRINITY_DN62922_c1_g4;TRINITY_DN63315_c2_g1;TRINITY_DN53828_c3_g3;TRINITY_DN78056_c1_g2;TRINITY_DN81533_c2_g2(down);TRINITY_DN78056_c1_g4;TRINITY_DN71122_c2_g3(down);TRINITY_DN67957_c1_g1;TRINITY_DN83040_c2_g1;TRINITY_DN53474_c0_g2;TRINITY_DN56561_c5_g2;TRINITY_DN78988_c1_g2;TRINITY_DN75563_c0_g1(down);TRINITY_DN78333_c0_g1;TRINITY_DN82458_c6_g1;TRINITY_DN82458_c6_g2;TRINITY_DN54659_c0_g1;TRINITY_DN55547_c1_g1;TRINITY_DN72217_c1_g2(down);TRINITY_DN52857_c0_g3;TRINITY_DN74339_c0_g1;TRINITY_DN82322_c1_g2(up);TRINITY_DN62070_c1_g3;TRINITY_DN78834_c1_g1(up);TRINITY_DN56694_c2_g1;TRINITY_DN61278_c0_g4(down);TRINITY_DN55906_c4_g3;TRINITY_DN67841_c2_g1;TRINITY_DN53686_c8_g3(down);TRINITY_DN80945_c2_g1;TRINITY_DN53900_c0_g1;TRINITY_DN82987_c3_g1(down);TRINITY_DN54449_c0_g1;TRINITY_DN81314_c2_g2;TRINITY_DN67731_c0_g2;TRINITY_DN60071_c3_g1;TRINITY_DN76379_c1_g1;TRINITY_DN62277_c0_g1;TRINITY_DN75969_c0_g1(down);TRINITY_DN57278_c0_g1;TRINITY_DN82498_c6_g3;TRINITY_DN83176_c2_g1(down);TRINITY_DN69798_c0_g4;TRINITY_DN48311_c0_g1(down);TRINITY_DN78389_c0_g1;TRINITY_DN48984_c1_g1;TRINITY_DN68004_c2_g2(up);TRINITY_DN78427_c3_g3;TRINITY_DN80178_c1_g1(down);TRINITY_DN83176_c1_g4;TRINITY_DN76680_c1_g3;TRINITY_DN54599_c2_g2;TRINITY_DN65491_c4_g1;TRINITY_DN82987_c2_g2;TRINITY_DN82690_c2_g2;TRINITY_DN82916_c0_g1;TRINITY_DN82690_c1_g1;TRINITY_DN77964_c0_g1;TRINITY_DN72231_c0_g2(up);TRINITY_DN82819_c3_g1;TRINITY_DN76407_c0_g3(down);TRINITY_DN78091_c0_g1;TRINITY_DN80761_c1_g1;TRINITY_DN77900_c0_g1(down);TRINITY_DN82596_c0_g1(down);TRINITY_DN75522_c0_g3;TRINITY_DN75522_c0_g1;TRINITY_DN70937_c1_g1;TRINITY_DN52530_c0_g1;TRINITY_DN82498_c6_g1(down);TRINITY_DN55384_c0_g1;TRINITY_DN82472_c2_g1;TRINITY_DN82068_c4_g1;TRINITY_DN70816_c0_g2;TRINITY_DN75821_c1_g1;TRINITY_DN66759_c1_g1;TRINITY_DN82650_c8_g2(down);TRINITY_DN82942_c4_g2(down);TRINITY_DN75164_c0_g1;TRINITY_DN75164_c0_g2;TRINITY_DN77976_c0_g1(down);TRINITY_DN66078_c2_g1;TRINITY_DN56242_c0_g1(down);TRINITY_DN70487_c3_g1	0.037190228
**ko00980**	**Metabolism of xenobiotics by cytochrome P450**	15 /2030	29 /5870	TRINITY_DN76573_c2_g2(up);TRINITY_DN78363_c1_g4;TRINITY_DN76630_c3_g2(up);TRINITY_DN69134_c0_g3(down);TRINITY_DN62216_c1_g2;TRINITY_DN78551_c1_g1(down);TRINITY_DN68184_c1_g1(down);TRINITY_DN58674_c0_g1;TRINITY_DN52148_c1_g1(up);TRINITY_DN62432_c1_g2;TRINITY_DN56447_c0_g1;TRINITY_DN78236_c4_g1(down);TRINITY_DN71180_c0_g3;TRINITY_DN71942_c1_g1;TRINITY_DN62432_c0_g1;TRINITY_DN83242_c16_g1(down);TRINITY_DN71746_c0_g1(down);TRINITY_DN62432_c1_g5(down);TRINITY_DN70523_c1_g1;TRINITY_DN62184_c0_g1;TRINITY_DN71411_c0_g1(up);TRINITY_DN62216_c1_g1(up);TRINITY_DN79688_c1_g2(down);TRINITY_DN74865_c1_g1;TRINITY_DN67605_c0_g1(up);TRINITY_DN31579_c0_g2;TRINITY_DN75691_c3_g3(down);TRINITY_DN60828_c0_g1;TRINITY_DN52014_c0_g1	0.04259601
ko04540	Gap junction	8 /2030	13 /5870	TRINITY_DN65297_c0_g2(down);TRINITY_DN82993_c3_g3(down);TRINITY_DN64491_c0_g1;TRINITY_DN82284_c5_g1;TRINITY_DN78211_c5_g2;TRINITY_DN70851_c2_g5;TRINITY_DN82083_c2_g1(down);TRINITY_DN51715_c0_g1;TRINITY_DN75784_c1_g3;TRINITY_DN70851_c2_g1;TRINITY_DN67309_c1_g2(down);TRINITY_DN81682_c1_g1(down);TRINITY_DN73850_c0_g2(down)	0.042897477
ko00944	**Flavone and flavonol biosynthesis**	3 /2030	4 /5870	TRINITY_DN77218_c1_g1(down);TRINITY_DN75819_c0_g2;TRINITY_DN71791_c0_g1(down);TRINITY_DN78634_c0_g1(down)	0.122450986
ko00942	Anthocyanin biosynthesis	1 /2030	1 /5870	TRINITY_DN72203_c2_g4	0.345826235
